# Sublethal Dose of *β*-Cypermethrin Impairs the Olfaction of *Bactrocera dorsalis* by Suppressing the Expression of Chemosensory Genes

**DOI:** 10.3390/insects13080721

**Published:** 2022-08-11

**Authors:** Shuang-Xiong Wu, Yang Chen, Quan Lei, Yuan-Yuan Peng, Hong-Bo Jiang

**Affiliations:** 1 Key Laboratory of Entomology and Pest Control Engineering, College of Plant Protection, Southwest University, Chongqing 400715, China; 2 International Joint Laboratory of China-Belgium on Sustainable Crop Pest Control, Academy of Agricultural Sciences, Southwest University, Chongqing 400715, China

**Keywords:** oriental fruit fly, sublethal effect, olfactory genes, pyrethroids

## Abstract

**Simple Summary:**

The oriental fruit fly, *Bactrocera dorsalis*, is a pest that causes huge economic losses in the fruit and vegetable industry. *β*-cypermethrin has been widely used in the orchard to control this major pest. According to a previous study, the oriental fruit fly developed significant resistance against *β*-cypermethrin in fields, which indicated that the oriental fruit fly has been exposed to sublethal concentrations of *β*-cypermethrin in the field for a long time. However, the sublethal effect and its mechanism are still unclear. In our present study, after treatment with sublethal concentrations of *β*-cypermethrin, the olfaction was disrupted significantly and the chemosensory genes were suppressed obviously. Our data demonstrated that the sublethal dose of *β*-cypermethrin impairs the olfaction of the pest insects by suppressing the expression of chemosensory genes, which provided theoretical guidance for the rational use of pesticides in fields.

**Abstract:**

The oriental fruit fly, *Bactrocera dorsalis*, is one of the most destructive fruit insect pests. *β*-cypermethrin has been widely used in the orchard to control this major insect. Based on the resistance monitoring in 2011, *B. dorsalis* developed significant resistance against *β*-cypermethrin in fields. This indicated that the *B. dorsalis* has been exposed to sublethal concentrations of *β*-cypermethrin in the field for a long time. Thus, it is urgent to understand the sublethal effects of *β*-cypermethrin on this fly to guide the rational use of an insecticide. According to the olfactory preference assays and electroantennogram (EAG) recording, the *B. dorsalis* after *β*-cypermethrin exposure (LD_30_ = 10 ng/fly) severely decreased the ability to perceive the tested odorants. Moreover, we then performed quantitative real-time PCR and found the chemosensory genes including odorant receptor co-receptor (*BdorORco*) and ionotropic receptor co-receptors (BdorIRcos) were obviously suppressed. Our results demonstrated that the sublethal dose of *β*-cypermethrin impairs the olfaction of the pest insects by suppressing the expression of chemosensory genes (*BdorORco* and BdorIRcos), which expanded our knowledge of the sublethal effects of the pesticide on insects.

## 1. Introduction

The oriental fruit fly, *Bactrocera dorsalis* (Hendel), attacks more than 600 fruit and vegetable crops [1,2]. It causes great economic losses through oviposition and larval feeding inside of the host plants [1,3]. Due to its rapid population growth, wide distribution and invasiveness, it has become one of the most destructive pests in tropical and subtropical countries [4,5]. Nowadays, chemical control is still the main method to control *B. dorsalis* and plays a significant role in the management of this fly [6,7,8]. *β*-cypermethrin, a pyrethroid, represents one of the major insecticides against *B. dorsalis* [5,7]. It behaves as a fast-acting neurotoxin in insects and it eventually leads to the death of target insects by prolonging the opening time of sodium ion channels [9,10].

In addition to the direct killing effect of insecticides on insects, a sublethal effect also existed in some individuals with different exposure amounts and times. It has been well documented that exposure to sublethal insecticides may cause multiple effects on the behavior, development and reproduction of insects [11,12]. On one hand, sublethal effects have negative effects on insects. For example, researchers found that a sublethal dose of indoxacarb and *β*-cypermethrin could significantly inhibit the growth and reproduction of *Rhopalosiphum padi* and *Plutella xylostella* and their offspring [9,13]. Exposure to a sublethal dose of deltamethrin detrimentally affects the reproduction and wing shape of *Chironomus columbiensis* [14]. Furthermore, spinosad exposure was found to impair the mobility of *Adoxophyes honmai* [15]. On the other hand, sublethal effects have “positive” effects on insects. The resurgence led by the hormesis effect of sublethal insecticides on the target insect pests is a great challenge to the rational use of pesticides [16]. For instance, exposure to sublethal concentrations of pesticides was also reported to stimulate the reproduction of several insects such as *Myzus persicae*, *Bemisia tabaci* and *Frankliniella occidentalis* [17,18,19].

Furthermore, an increasing number of studies have shown that sublethal doses of insecticides can severely damage olfaction, resulting in abnormal olfactory behaviors, especially in non-target organisms [20]. Currently, the sublethal effects of insecticides on non-target pests have become an interesting topic in the pesticide environmental toxicology study [20]. In *Oncorhynchus mykiss*, the sublethal dose of chlorpyrifos impaired olfactory signal transduction resulting in olfaction sensitivity disruption [21]. Moreover, the dose-dependent sublethal concentration of the imidacloprid impaired host finding and sexual communication in *Nasonia vitripennis* [22]. After exposure to a sublethal dose of imidacloprid, the nervous system in the calyces regions responsible for both olfaction and vision was damaged, causing decreased olfaction learning ability in *Apis mellifera* [23].

In 2011, the resistance monitoring of *B. dorsalis* in mainland China showed that 24 out of the 25 field populations developed different resistance against *β*-cypermethrin ranging from 3.0 folds to 44.0 folds [7]. Due to the rapid development of the resistance, *B. dorsalis* has probably been exposed to sublethal concentrations of *β*-cypermethrin in fields since 2011. However, the sublethal effect of *β*-cypermethrin on *B. dorsalis* still remains unknown, especially whether there are any significant effects on its olfaction. Therefore, we conducted the olfactory behavior and electroantennogram (EAG) assays to investigate the disruption of the olfaction in *B. dorsalis* exposed to a sublethal dose of *β*-cypermethrin. Moreover, to figure out its potential mechanism, we then performed quantitative real-time PCR to determine the expression profiles of chemosensory genes including *BdorORco* and BdorIRcos. The results not only demonstrated that the sublethal dose of *β*-cypermethrin impairs the olfaction of *B. dorsalis* by suppressing the expression of chemosensory genes (*BdorORco* and BdorIRcos), but also provided insights into the physiological effects of *β*-cypermethrin on the target insect pest besides its insecticidal effects. Our data will also provide a reference for the scientific use of pesticides in pest management in fields.

## 2. Materials and Methods

### 2.1. Insects

The *B. dorsalis* population used in our experiments was originally collected from Hainan province, China, in 2008. All life stages of the oriental fruit flies were kept in a growth chamber at 27.5 ± 1 °C, 75 ± 5% relative humidity, with a 14 h light:10 h dark photoperiod [24]. Adults were fed an aqueous artificial diet of yeast powder, honey, sugar and vitamin C [25]. Male and female adults were separated within 3 days of the eclosion. All the adults were synchronized under the same conditions before experimentation.

### 2.2. β-Cypermethrin Bioassay

*β*-cypermethrin (96% purity) was provided by the Institute for Control of Agrochemicals, Sichuan province, China. The topical application method was used in the bioassay [26]. A serial dilution of *β*-cypermethrin in acetone (0, 5 ng/fly, 10 ng/fly, 15 ng/fly, 20 ng/fly, 25 ng/fly) was applied to the pronotum of 6-day-old flies, using a PB600-1 repeating dispenser (Hamilton, Reno, NV, USA). Flies applied with acetone served as a control. Mortality was recorded at 24 h after *β*-cypermethrin treatment. There were three biological replications with 60 flies for each.

### 2.3. Olfactory Preference Assays

The four choice olfactometer assay was carried out to detect the olfactory preference of treated flies (*β*-cypermethrin, 10 ng/fly) to 1-octen-3-ol (Sigma-Aldrich, St Louis, MO, USA), methyl eugenol (Acros, Morris Plains, NJ, USA) and ethyl acetate (Sigma, St Louis, MO, USA) followed the published method [27] with slight modification. The apparatus consisted of a central chamber connected to four 50 mL cylindrical glass bottles. Two of the glass bottles at the opposite corner were designated for odor stimuli treatments and the other two for controls. Purified air at a rate of 200 mL/min was drawn towards the center of the olfactometer simultaneously from each arm and removed via the vacuum pump at the rate of 1000 mL/min.

1-octen-3-ol was regarded as a female attractant [28], while methyl eugenol was a male-specific attractant [29]. Meanwhile, ethyl acetate was proved to attract both male and female flies [30]. Therefore, only females were used for the assay of 1-octen-3-ol, while only males were used for the assay of methyl eugenol. Meanwhile, both females and males with a sex ratio of 1:1 were employed in the assay of ethyl acetate. Each glass bottle was filled with a quarter piece of filter paper (Newstart, Hangzhou, China) that was impregnated with 100 μL of diluted odorant in the concentration of 1% (*v*/*v*) or control. In detail, the control for 1-octen-3-ol and ethyl acetate is mineral oil (MO, Sigma-Aldrich, St Louis, MO, USA), and the control for methyl eugenol is dimethyl sulfoxide (DMSO, Sigma-Aldrich, St Louis, MO, USA). Twenty 7-day-old flies (after treatment) were placed in the center of the olfactometer and continuously monitored for 10 min. Each group contained 6 replicates. Flies that stayed at the center of the apparatus longer than 10 min were considered “non-responders”. The attraction rate was calculated by dividing the number of flies attracted per treatment by the total number of flies entering the olfactometer. In addition, the *B. dorsalis* were tested at the same time point to guarantee the consistent rhythm.

### 2.4. Electroantennogram (EAG)

The EAG responses of the *β*-cypermethrin treated (LD_30_ = 10 ng/fly) flies were measured with a standard protocol [31]. Briefly, the whole head of the 7-day-old fly (after treatment) was excised and connected to two glass micropipettes filled with 0.9% NaCl. One glass micropipette was in contact with the distal tip of the antenna as the recording electrode, the other one was inserted into the base of the head, which served as the reference electrode. The response signal was recorded using EAG-2000 software (Syntech, Hilversum, The Netherlands) with an IDAC4 amplifier (Syntech, Hilversum, The Netherlands). A controller generated 100 mL/min of pulse flow to stimulate the fly’s antenna.

Three plant volatiles (1-octen-3-ol, methyl eugenol and ethyl acetate) were selected for EAG experiments [28,29,30]. In detail, only females were used for the assay of 1-octen-3-ol, while only males were used for the assay of methyl eugenol. Meanwhile, both females and males with a sex ratio of 1:1 were employed in the assay of ethyl acetate. As described above, MO or DMSO was used as the solvent and the negative control. Ten microliters of each dilution or control were applied to a paper strip (5 × 1 cm) which served as the stimulus source. Each stimulation lasted 1 s, and stimulus interval was at least 30 s [31]. There were about 10 individuals for the EAG assay of each plant’s volatile.

### 2.5. Quantitative Real-Time PCR

The flies were treated with acetone or *β*-cypermethrin (LD_30_ = 10 ng/fly) at 6 days old. After 24 h, their heads were dissected and immediately frozen in liquid nitrogen. In detail, the samples of males and females were separately dissected and mixed in equal proportions. Total RNA was extracted from the collected samples with TRIzol (Invitrogen, Carlsbad, CA, USA). First-strand cDNA was synthesized using 1µg of total RNA and PrimeScript 1st Strand cDNA Synthesis Kit (Takara, Dalian, China).

A standard protocol [32] was applied in this study, and the amplification efficiencies of primers were validated by generating a standard curve based on a double-fold cDNA dilution series. The 10 μL qPCR system consisted of 5 µL of SYBR Supermix (Novoprotein, Shanghai, China), 10 pM of the specific primers of each gene, 250 ng cDNA and nuclease-free water. The qPCR reaction was conducted on a CFX Conncet^TM^ Real-Time System (Bio-Rad, Jurong, Singapore) with the following program: 2 min at 95 °C for pre-denaturation, followed by 40 cycles of 95 °C for 15 s and 60 °C for 30 s. A melting curve analysis generated from 60 °C to 95 °C was used to verify the specificity and consistency of the products for each primer pair [33]. *α-tubulin* (GenBank: GU269902) and *ribosomal protein S3* (*rps 3*, GenBank: XM_011212815) were selected as the internal reference genes. All the primers used in this study were designed by Primer Premier 5 (PREMIER Biosoft International, Palo Alto, California, USA) as shown in Table 1. Four biological and two technical replicates were set for each experiment. The relative expression data were analyzed using the 2^−^^ΔΔCt^ method [34].

### 2.6. Statistical Analysis

All experiments were performed with at least three biological replications, and all the data were analyzed with SPSS software version 25.0 for Windows (IBM, Chicago, IL, USA). The concentration-mortality data of *β*-cypermethrin bioassay were analyzed by probit analysis. The results of olfactory preference assays, EAG recording and chemosensory genes relative expression after *β*-cypermethrin induction were analyzed with Student’s *t*-test (* *p* < 0.05; ** *p* < 0.01; *** *p* < 0.001).

## 3. Results

### 3.1. Bioassay

We applied *β*-cypermethrin to the pronotum of cold-sedated *B. dorsalis* in 6-day-olds. The mortality for the tested concentrations was assessed 24 h after the *β*-cypermethrin application, which ranged from 11.7% to 78.3%. Based on the probit analysis, the dose causing 50% mortality (LD_50_) within a 24 h observation period was 14.50 ng per fly (Table 2). According to the calculation, the LD_30_ would be 9.70 ng/fly. Thus, we used this sublethal dose for the subsequent study.

### 3.2. Olfactory Preference

**Figure 1:**
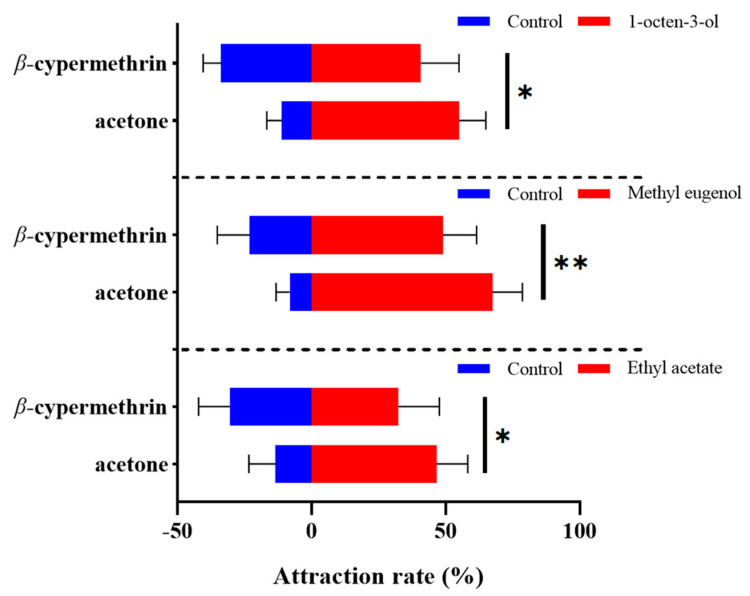
Four-way olfactometer assay of 7-day-old adults treated by *β*-cypermethrin (LD_30_ = 10 ng/fly). We used 1-octen-3-ol, methyl eugenol and ethyl acetate at 1% (*v*/*v*) concentration as the attractants. Females, males and both sexes were employed in the assays of 1-octen-3-ol, methyl eugenol and ethyl acetate, respectively. Data were presented as mean ± SE (*n* = 6). Asterisks represent a significant difference determined by Student’s *t*-test (* *p* < 0.05; ** *p* < 0.01).

The alteration in olfactory preference of the 7-day-old flies treated with *β*-cypermethrin was determined by an olfactory behavior assay using the four-choice olfactometer. Three plant volatiles including 1-octen-3-ol, methyl eugenol and ethyl acetate were used at the concentration of 1% (*v*/*v*). As the results indicated, the *B. dorsalis* exposed to sublethal doses of *β*-cypermethrin significantly disrupted the discernment of the tested odorant (Figure 1). After *β*-cypermethrin treatment, the attraction rate of 1-octen-3-ol to the females decreased by 14.3% (from 55.0% to 40.7%), while the attraction rate of methyl eugenol to the males decreased by 18.5% (from 67.4% to 48.9%). Furthermore, the attraction rate of ethyl acetate to both females and males (with a sex ratio of 1:1) decreased by 14.2% (from 46.6% to 32.4%), specifically. Notably, there was no significant difference between the male and female flies in the ethyl acetate attraction assay.

### 3.3. EAG Analysis

**Figure 2:**
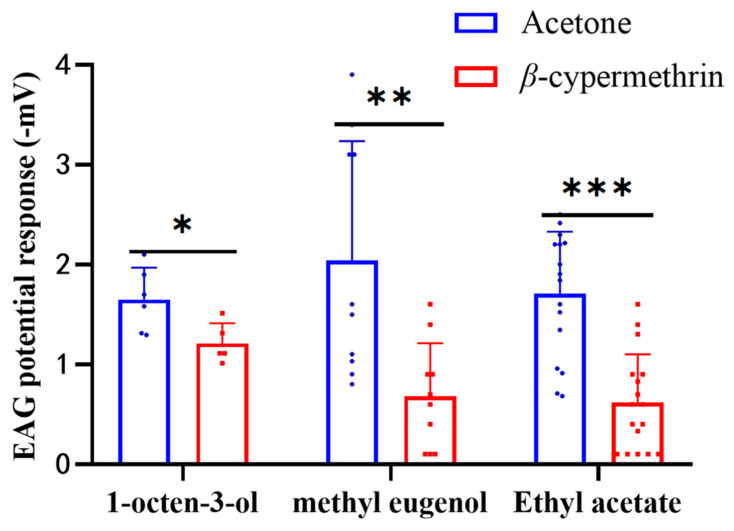
EAG recording of 7-day-old flies treated by *β*-cypermethrin (LD_30_ = 10 ng/fly). The EAG data was obtained by stimulating flies with three odorants including 1-octen-3-ol, methyl eugenol and ethyl acetate at 1% (*v*/*v*) concentration. Ethyl acetate and 1-octen-3-ol were diluted with MO and methyl eugenol was diluted with DMSO. Females, males and both sexes were employed in the assays of 1-octen-3-ol, methyl eugenol and ethyl acetate, respectively. Data were presented as mean ± SE, and asterisks represent a significant difference with analysis of Student’s *t*-test (*n* = 8–12, * *p* < 0.05; ** *p* < 0.01; *** *p* < 0.001).

To study the electrophysiological response of *B. dorsalis* treated with *β*-cypermethrin, the EAG responses of 7-day-old adult flies to 1% (*v*/*v*) concentration of 1-octen-3-ol, methyl eugenol and ethyl acetate were recorded (Figure 2). The EAG signal ranged from −0.6 to −3.5 mV for 1-octen-3-ol, −0.6 to −3.9 mV for methyl eugenol, and −0.3 to −3.3 mV for ethyl acetate. However, the EAG responses of the flies exposed to *β*-cypermethrin were significantly weaker than the control for all three odorants. After *β*-cypermethrin treatment, the EAG response of the females to 1-octen-3-ol was reduced by 26.6%, while the EAG response of the males to methyl eugenol was reduced by 66.7%. In addition, the EAG response of both females and males (with a sex ratio of 1:1) to ethyl acetate was reduced by 63.9%. Notably, there was no significant difference between the male and female flies in the EAG assay of ethyl acetate.

### 3.4. Differential Expression of Olfactory Genes upon β-Cypermethrin Exposure

**Figure 3:**
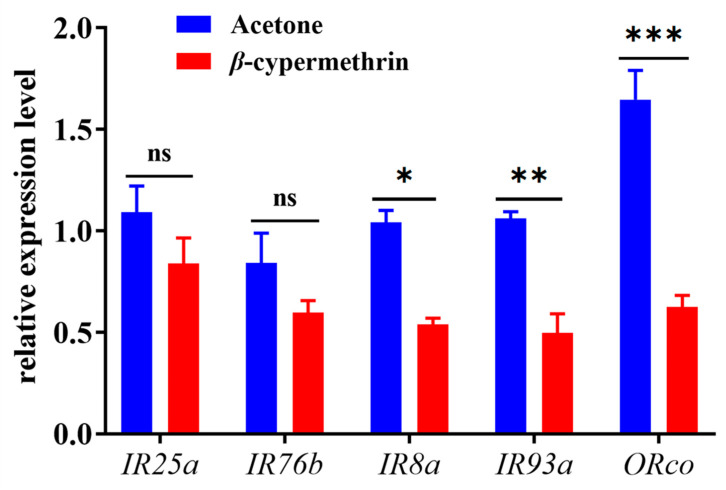
Transcriptional expression profiles of *ORco* and IRcos after *β*-cypermethrin induction (LD_30_ = 10 ng/fly). Data were presented as mean ± SE, and asterisks represent a significant difference with analysis of Student’s *t*-test (*n* = 4, *p < 0.05; **p < 0.01; ***p < 0.001). No significant difference was represented by “ns”.

The relative expressions of chemosensory genes in the 7-day-old adult flies under *β*-cypermethrin treatment were analyzed by RT-qPCR (Figure 3). The amplification efficiencies of *α-tubulin*, *rps 3*, *BdorORco*, *BdorIR8a*, *BdorIR25a*, *BdorIR76b* and *BdorIR93a* were 97.5%, 98.3%, 95.3%, 97.7%, 96.5%, 101.5% and 90.7%, respectively (Table 1). As the results indicated, a sublethal dose of *β*-cypermethrin impaired the expression of chemosensory genes in *B. dorsalis*. Compared with the control group, the expression of *BdorIR8a*, *BdorIR93a* and *BdorORco* of *β*-cypermethrin treated flies significantly decreased by 48.2%, 53.3%, and 62.1%, respectively. However, the relative expression of *IR25a* and *IR76b* after treatment had no significant differences.

## 4. Discussion

The oriental fruit fly has probably been exposed to sublethal concentrations of insecticides in fields for a long time, since the resistance monitoring of *B. dorsalis* in mainland China showed that only 1 of the 25 populations stay sensitive to *β*-cypermethrin [7]. Therefore, it is necessary to investigate the sublethal effects of *β*-cypermethrin on this fly. Compared with the susceptible strain described in the previous study, the *B. dorsalis* strain in our laboratory possessed a medium resistance with a resistance ratio of 11.7 (based on the LD_50_) to *β*-cypermethrin [7]. Thus, we exposed the flies to *β*-cypermethrin with the dose of LD_30_ (10 ng/fly) in this study.

Sublethal insecticide residues have been shown to cause multiple effects on insects. For example, *Bacillus thuringiensis* exposure was found to reduce the lifespan and reproductive capacity of *Helicoverpa armigera* [35]. In *Cyrtorhinus lividipennis*, researchers found that sublethal concentration of triazophos and deltamethrin disturbed foraging ability, reduced predatory capacity, and decreased pepsin activity [36]. Compared with the susceptible strain, the cyantraniliprole-resistant (19.44 folds) strain of *B. dorsalis* shows a prolonged larval duration, higher pupa weight, and longer timing of sexual maturation [37]. Interestingly, a very low-dose exposure of chlordimeform induced an increased sensitivity to sex pheromone in *Carposia niponensis* [38]. Likewise, exposure to a sublethal dose of *β*-cypermethrin also improved the abilities of motility and respiration in *Harmonia axyridis* [39]. In the current study, *B. dorsalis* exposed to a sublethal dose of *β*-cypermethrin showed an altered olfactory behavior. However, we do not know whether development and reproduction were influenced or not. Moreover, we do not know whether the *β*-cypermethrin residue resulted in hormesis effects on this fly either. Hence, future studies on its developmental biology and ecological fitness are needed to investigate whether the sublethal effects of *β*-cypermethrin are contributing to the resurgence of *B. dorsalis* in a field or not.

The olfactory system of *B. dorsalis* is essential for finding habitats and mates, foraging, ovipositing, and avoiding predators [40,41,42]. Olfactory reception in *B. dorsalis* is represented by two main types of molecular receptors, the odorant receptors (ORs) and the ionotropic receptors (IRs) [43]. Recently, studies have shown that sublethal doses of insecticides can seriously damage insect olfaction, resulting in abnormal olfactory behaviors. Our olfactory preference assays and EAG recording indicated that the *B. dorsalis* after *β*-cypermethrin exposure (LD_30_ = 10 ng/fly) severely decreased the ability to perceive the tested odorants. We are aware that we tested the females and males separately either for the behavior or the EAG for 1-octen-3-ol and methyl eugenol, due to the characterizations of the tested odorants. However, we concluded that the impaired olfaction by a sublethal dose of *β*-cypermethrin is not sex-biased, since there was no significant difference between sexes in the assays for ethyl acetate. Moreover, our results are consistent with several previous studies. In *N. vitripennis*, sublethal doses of the imidacloprid damaged the olfaction thereby impairing sexual communication and host finding [22]. In *Spodoptera littoralis*, the sublethal dose of deltamethrin residue reduced the repolarization time to sex pheromone and host–plant odorants and overexpresses some detoxification metabolic enzyme genes [44]. It would be very interesting to further study whether the olfactory alteration contributed to the development of resistance in insects or not.

According to the previous studies, a sublethal dose of chlorpyrifos impaired olfactory signal transduction resulting in olfaction sensitivity disruption in *O. mykiss* [21]. Besides, in *A. mellifera*, the olfaction learning ability decreased after exposure to a sublethal dose of imidacloprid because the density of the synaptic units in the region of the calyces that are responsible for olfactory and visual functions decreased [23]. In *Agrotis ipsilon*, sublethal doses of clothianidin residue decreased behavioral sex pheromone responses by downregulating the sensitivity of antennal lobe output neurons [45]. In the present study, according to the EAG and qPCR data, we found that the impaired olfaction may be due to the significantly reduced expression of chemoreceptor genes closely related to olfaction. This is consistent with the result that a sublethal dose of imidacloprid suppresses the *ORco* and *IR8a-2* in *Aphidius gifuensis* by analyzing transcriptome [46]. As our qPCR results indicated, the relative expression of *ORco*, *IR8a* and *IR93a* after treatment were significantly lower than control while the relative expression of *IR25a* and *IR76b* after treatment had no significant differences. Hence, we speculated that *IR8a* and *IR93a* are more closely associated with olfaction rather than *IR25a* and *IR76b* which have a more widespread expression (undocumented) [47,48]. However, due to the limitation of technical conditions, we do not know whether insecticide residue impairs the sensilla present in various tissues such as antennae and maxillary palp, olfactory signal transduction system, or the nervous system, which is worthy of further study.

In general, our data suggested that it might be difficult for *B. dorsalis* to proliferate and invade under sublethal *β*-cypermethrin stress. However, when non-target insects other than *B. dorsalis* encounter *β*-cypermethrin stress in the field, their olfaction is most likely affected. Further study on the ecological and environmental toxicology of sublethal dose of *β*-cypermethrin on non-target insects will provide answers for the effects on their populations. Therefore, our data not only expanded the knowledge on the sublethal effects of insecticides but also provided theoretical guidance for the rational use of pesticides in fields.

## Figures and Tables

**Table 1:** Primer sequences of chemosensory genes used for quantitative real-time PCR.

Primer	Sequence (5′-3′)	Amplification Efficiency	Product Length (nt)
qPCR-*α-tubulin*-F	CGCATTCATGGTTGATAACG	97.5%	184
qPCR-*α-tubulin*-R	GGGCACCAAGTTAGTCTGGA		
qPCR-*rps 3*-F	TAAGTTGACCGGAGGTTTGG	98.3%	169
qPCR-*rps 3*-R	TGGATCACCAGAGTGGATCA		
qPCR-*ORco*-F	TTGACATCCACCATTATGCTGAC	95.3%	209
qPCR-*ORco*-R	TCCTCGGAGCCATCATACCA		
qPCR-*IR8a*-F	ATTGCGGCGTTGGTGGGTA	97.7%	185
qPCR-*IR8a*-R	GAGACGGCTTTTGGTGCTT		
qPCR-*IR25a*-F	TTGCTCCAGGTAATGCCTCC	96.5%	189
qPCR-*IR25a*-R	TCGTTTTCCCTCCTTCGCAA		
qPCR-*IR76b*-F	CCACTTTGGACGAGGGTGAA	101.5%	194
qPCR-*IR76b*-R	AGGCTTCTGCTCCTTATCGC		
qPCR-*IR93a*-F	AAGTGTAGCGGTCATGGTGG	90.7%	171
qPCR-*IR93a*-R	TGCAAAGACACCTCGCTTCT		

**Table 2:** Toxicity of *β*-cypermethrin against 6-day-old adults of *B.dorsalis*.

Insecticide	*n*	Slope ± SE	X^2^	df	Concentration (95% CI) (ng/fly)	RR *
*β*-cypermethrin	360	2.85 ± 0.47	1.96	4	LD_30_ = 9.70 (7.91–11.18)	11.7
					LD_50_ = 14.50 (12.76–14.46)	

* Resistance ratios: LD_50_ divided by LD_50_ of susceptible strain.

## Data Availability

All data are available in this paper.
